# Resolution of Conflicting Signals at the Single-Cell Level in the Regulation of Cyanobacterial Photosynthesis and Nitrogen Fixation

**DOI:** 10.1371/journal.pone.0066060

**Published:** 2013-06-21

**Authors:** Wiebke Mohr, Tomas Vagner, Marcel M. M. Kuypers, Martin Ackermann, Julie LaRoche

**Affiliations:** 1 Department of Biogeochemistry, Helmholtz Centre for Ocean Research (GEOMAR), Kiel, Germany; 2 Department of Biogeochemistry, Max Planck Institute for Marine Microbiology, Bremen, Germany; 3 Department of Environmental Systems Science, Swiss Federal Institute of Technology, Zürich, Switzerland; 4 Department of Environmental Microbiology, Eawag, Dübendorf, Switzerland; University Paris South, France

## Abstract

Unicellular, diazotrophic cyanobacteria temporally separate dinitrogen (N_2_) fixation and photosynthesis to prevent inactivation of the nitrogenase by oxygen. This temporal segregation is regulated by a circadian clock with oscillating activities of N_2_ fixation in the dark and photosynthesis in the light. On the population level, this separation is not always complete, since the two processes can overlap during transitions from dark to light. How do single cells avoid inactivation of nitrogenase during these periods? One possibility is that phenotypic heterogeneity in populations leads to segregation of the two processes. Here, we measured N_2_ fixation and photosynthesis of individual cells using nanometer-scale secondary ion mass spectrometry (nanoSIMS) to assess both processes in a culture of the unicellular, diazotrophic cyanobacterium *Crocosphaera watsonii* during a dark-light and a continuous light phase. We compared single-cell rates with bulk rates and gene expression profiles. During the regular dark and light phases, *C. watsonii* exhibited the temporal segregation of N_2_ fixation and photosynthesis commonly observed. However, N_2_ fixation and photosynthesis were concurrently measurable at the population level during the subjective dark phase in which cells were kept in the light rather than returned to the expected dark phase. At the single-cell level, though, cells discriminated against either one of the two processes. Cells that showed high levels of photosynthesis had low nitrogen fixing activities, and vice versa. These results suggest that, under ambiguous environmental signals, single cells discriminate against either photosynthesis or nitrogen fixation, and thereby might reduce costs associated with running incompatible processes in the same cell.

## Introduction

Dinitrogen (N_2_) fixation and photosynthesis are two crucial metabolic processes in diazotrophic cyanobacteria. However, cyanobacterial photosynthesis leads to the production of O_2_, which inactivates the key enzyme for N_2_ fixation, nitrogenase [1], [2]. The two processes can thus not be performed concurrently within one cell. To overcome this problem, cyanobacterial diazotrophs separate N_2_ fixation and photosynthesis, either spatially or temporally. The development of specialized cells in filamentous cyanobacteria provides a spatial separation, with N_2_ fixation occurring in the thick-walled heterocysts that lack the oxygenic photosystem (PS) II. Unicellular cyanobacteria separate N_2_ fixation and photosynthesis temporally, with the former occurring during the dark and the latter in the light [3]–[6]. This segregation of N_2_ fixation and photosynthetic activity is regulated by a circadian clock [4], [7]–[9]. Cyanobacteria possess the simplest version of a circadian regulatory network with the key proteins encoded by the *kaiABC* genes (for review see [10]). A recent study suggests that this circadian clock is directly entrained by light-driven changes in energy metabolism [11]. Since the circadian clock controls the expression of the nitrogenase, the direct entrainment of the clock by light leads to a temporal separation of the two processes.

However, this separation may not always be complete. Several studies suggest that photosynthesis and nitrogen fixation could occur concurrently, at least on the population level, during transitions from dark to light [12] or in studies where the light conditions are experimentally shifted relative to the clock [13]. However, both studies did not directly measure N_2_ fixation and photosynthesis at the single-cell level leaving the question whether individual cells actually do perform both processes. There is evidence that some populations have the capacity to adjust their cellular metabolism in order to fix N_2_ and CO_2_ simultaneously after acclimation to continuous light [6]. So far, this observation appears to be restricted to the unicellular diazotrophic cyanobacteria *Gloeothece* sp. [6], [14] and *Synechococcus* sp. RF-1 [15]. Recently, it has also been observed that the unicellular diazotrophic cyanobacterium *Crocosphaera watsonii* was able to grow diazotrophically in continuous light, however, the question remains whether this involves simultaneous or time-resolved fixation of N_2_ and CO_2_ [16].

These results raise the question of how individual cells manage the activities of the two processes under such conditions. Does the observation of photosynthesis and nitrogen fixation at the population level mean that each cell performs these processes simultaneously – or do single cells preferentially perform one or the other process thereby indicating phenotypic heterogeneity between genetically identical cells of a population? A number of recent studies have reported on the latter, for diverse phenotypic traits including behavior [17] growth rate [18], [19], and gene expression [20].

N_2_ fixation of single cells has already been analyzed in previous studies including field studies [21]; for example, *Teredinibacter turnerae* was shown to exhibit population heterogeneity in N_2_ fixation [22] whereas vegetative cells in *Anabaena oscillarioides* filaments had a rather uniform distribution of recently fixed nitrogen from the heterocysts [23]. The former is a heterotrophic proteobacterium which is diazotrophic but not photosynthetic, and the latter is a heterocystous cyanobacterium which fixes N_2_ and is photosynthetic but has the capability of spatially separating N_2_ fixation and photosynthesis. In contrast, *C. watsonii* is a unicellular diazotrophic cyanobacterium that can only separate the two processes temporally, indicating the need for a tight temporal regulation such as a circadian clock [13].

Here, we combined the measurement of bulk rates of N_2_ fixation and photosynthesis in a population of *C. watsonii* WH8501 with single-cell measurements using nanoSIMS throughout dark-light and continuous light phases. We complemented rate measurements with the analysis of gene expression patterns at the population level for genes that are involved in these two processes. This allowed us to assess the level of phenotypic heterogeneity during regular light-dark cycles, and under conditions where populations that are ‘scheduled’ to perform O_2_-sensitive N_2_ fixation are exposed to light, and thus potentially are subject to a physiological dilemma posed by the opportunity to simultaneously carry out two incompatible processes.

## Results and Discussion

### N_2_ Fixation and Photosynthesis at the Population Level

**Figure 1:**
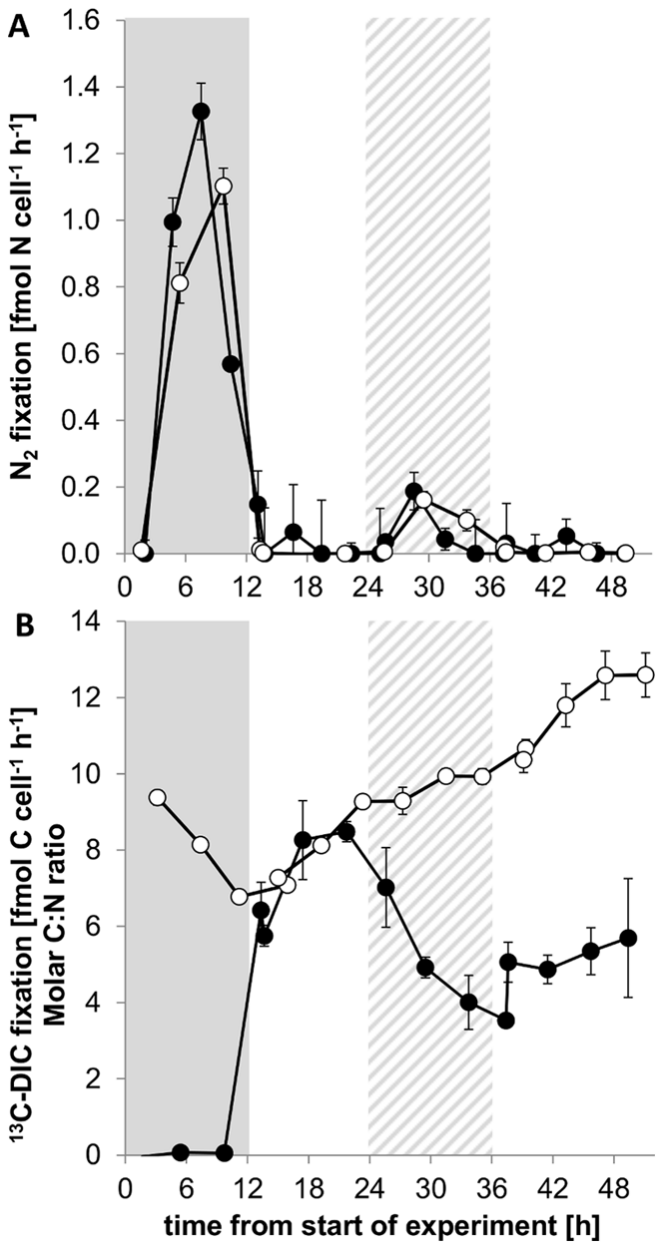
N_2_ fixation and photosynthesis during the dark-light and subjective dark-light phases. A. N_2_ fixation measured via acetylene reduction assay (ARA; filled circle) and ^15^N_2_ incubation (open circle). B. Photosynthesis measured via NaH^13^CO_3_ incubation (filled circle) and molar C:N ratio (open circle). The grey bars indicate the regular dark phase and the striped grey bars indicate the subjective dark phase. Symbols and error bars represent mean ± SE of triplicate cultures.

N_2_ fixation rates in *C. watsonii* determined via the acetylene reduction assay (ARA) followed the diel pattern known for this organism [24], [25] during the first 24 h of the experimental phase encompassing a 12∶12 h dark:light cycle with N_2_ fixation occurring during the dark phase (Figure 1A). N_2_ fixation rates measured via the incorporation of ^15^N_2_ into biomass (Figure 1A) closely matched the observed reduction of acetylene with a ratio of 4.5∶1 of ethylene produced to N_2_ fixed, which compared well with the conversion factor of 4∶1 [26] (ratio from total dark N_2_ fixation). Inorganic carbon fixation was confined to the light phase of the dark:light cycle (Figure 1B), in line with the well-established temporal separation of N_2_ fixation and photosynthesis in unicellular, diazotrophic cyanobacteria [2], [3], [5], [6].

We then exposed *C. watsonii* to a 24 h continuous light period just after a 12∶12 h dark:light cycle to determine how this bacterium regulates N_2_ fixation and photosynthesis considering the dilemma that continuous light poses on the population. Unexpectedly, both inorganic carbon and N_2_ fixation rates were measurable concurrently at the population level (Figure 1) during the subjective dark phase in which the cells were kept in continuous light rather than returned to the expected dark phase. However, N_2_ fixation only reached 14% of the maximum observed during the regular dark phase. Photosynthetic rates during the subjective dark phase continuously declined to 42% of its maximum regular light phase activity at the end of the subjective dark period. This indicates that photosystem components are not entirely degraded at the “scheduled” end of the light period and can be activated upon illumination [25]. The relative rates of carbon and nitrogen accumulation in the biomass during the subjective dark phase are reflected in the dynamics of the molar C:N (carbon:nitrogen) ratio of the biomass (Figure 1B). The increase in C:N decelerates compared to the previous light phase, but is still significantly positive (regression analysis; p<0.05). Taking into account the measured rates of N_2_ fixation and photosynthesis as well as the molar C:N ratio of the biomass, about 67% of this deceleration could be attributed to the sustained but lower rates of photosynthesis and N_2_ fixation. We attribute the other 33% to additional respiration, which may have supported nitrogenase activity in the light [27].

**Figure 2:**
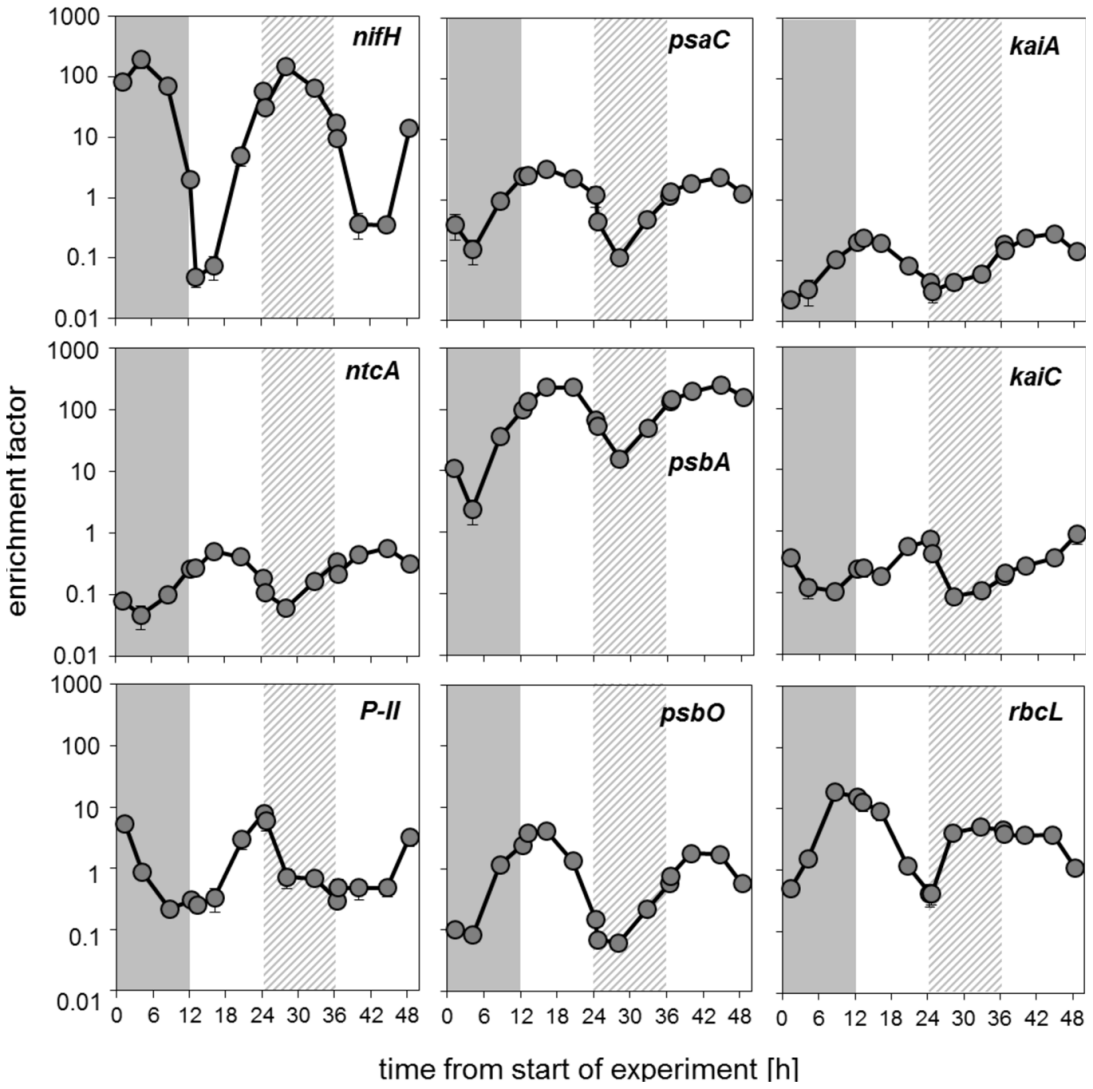
Gene expression analysis shown as enrichment factor of relative transcript abundance. The genes are indicated in the top right corner of each panel. Filled circles represent the experimental data during the 48 h phase. The grey bars indicate the regular dark phase and the striped grey bars indicate the subjective dark phase. Symbols and error bars represent mean ± SE of triplicate cultures.

We then measured the expression of genes related to N_2_ fixation and photosynthesis in order to analyze how the co-occurrence of N_2_ fixation and photosynthetic activity during subjective dark would manifest at the transcriptional level. Interestingly, expression levels of *nifH* did not show any differences in peak activity between the regular and the subjective dark phase (Figure 2). This indicates that the reduction of N_2_ fixation in the subjective dark phase may have been regulated post-transcriptionally [28], possibly resulting from the inactivation of the nitrogenase complex by photosynthetically evolved O_2_. All genes analyzed here showed cyclic patterns comparable to those found in the regular dark and light phases (Figure 2) suggesting circadian regulation of N_2_ fixation and photosynthesis in *C. watsonii* [13]. We also analyzed two genes coding for proteins of the cyanobacterial circadian clock, *kaiA* and *kaiC*. Both genes showed cyclic expression patterns throughout the entire experimental phase giving further evidence for circadian regulation in which cyclic gene expression is maintained for several cycles in continuous light (e.g. [29]).

### N_2_ Fixation and Photosynthesis at the Single-cell Level

**Figure 3:**
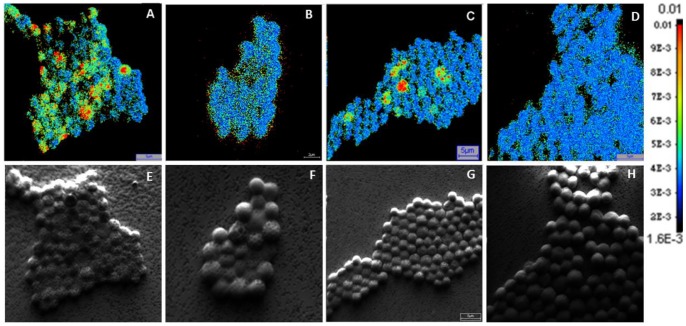
Enrichment in ^15^N (color scale: ^15^N/^14^N) due to N_2_ fixation by individual *C.* *watsonii* cells. A. Regular dark phase. B. Regular light phase. C. Subjective dark phase. D. Subjective light phase. (Scale bars: 5 µm in A, C and D, 2 µm in B). E–H. Secondary electron images (complementary to A–D.) which are simultaneously recorded during the measurement and showing the individual cells. The aggregation of cells was an artifact of filtration; the *C. watsonii* cells are unicellular (as per microscopic observation), however, gather in trenches upon filtration due to the unevenness of the filtration devices at the micrometer scale.

**Figure 4:**
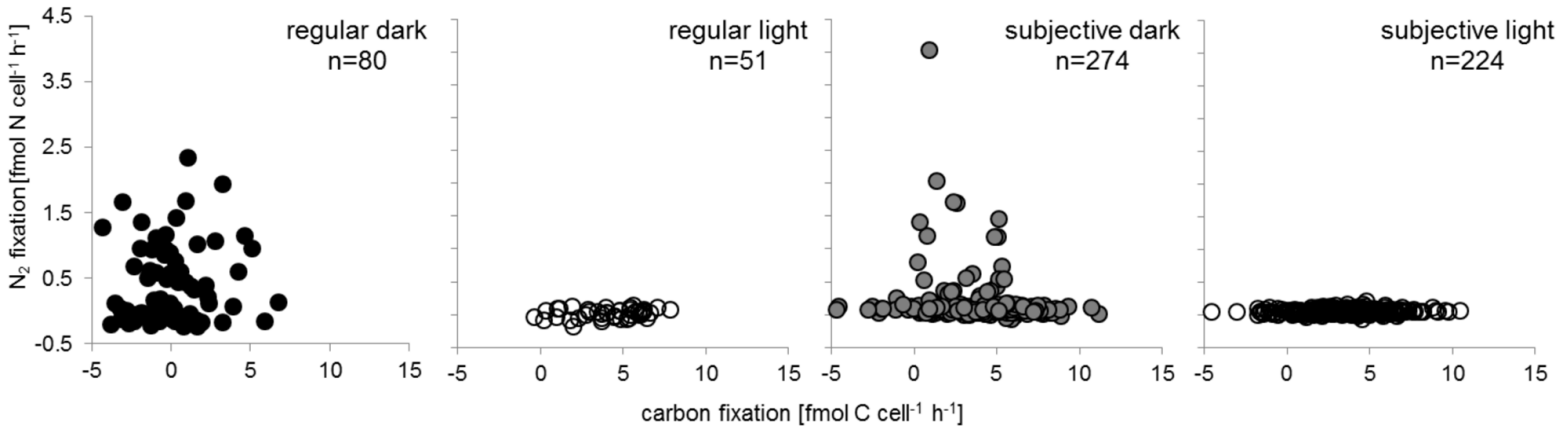
^15^N_2_ fixation and photosynthesis (NaH^13^CO_3_ uptake) rates as calculated from the isotopic enrichment of individual cells (each symbol represents one individual cell). The corresponding dark or light phase is indicated in the upper right corner of each panel. The large variability in the ^13^C signal/photosynthesis for the regular dark phase is due to the precision of the nanoSIMS measurement combined with the low labeling during the non-photosynthetic phase.

In general, the single-cell analysis using nanoSIMS revealed the same temporal pattern of N_2_ fixation and photosynthetic activity during the regular light-dark cycle as the bulk population measurements (Figure 1). Cells fixed N_2_ during the regular dark period with no dissolved inorganic carbon (DIC) uptake, and photosynthesized with no N_2_ fixation during the regular light period (Figures 3 and 4, Table 1).

**Table 1:** Summary of single-cell photosynthesis (DIC uptake) and N_2_ fixation rates.

DIC uptake	(regular dark)*	(regular light)	(subjective dark)	(subjective light)
range	−5.09–6.78	−0.34–11.23	−4.70–11.17	−4.58–10.44
mean ± SD	0.19±2.34	4.61±2.51	3.75±2.55	3.44±2.61
median	−0.05	4.79	3.96	3.62
N_2_ fixation	(regular dark)†	(regular light)‡	(subject. dark)†	(subject. light)‡
range	−0.23–2.35	−0.19–0.14	−0.07–4.03	−0.06–0.20
mean ± SD	0.40±0.60	0.02±0.07	0.17±0.37	0.05±0.03
median	0.13	0.03	0.09	0.05

Values are in fmol C and N cell^−1^ h^−1^ for DIC uptake and N_2_ fixation, respectively, and represent mean ± SD.

* significantly different from all other phases, all other phases are not significantly different from each other.

† not significantly different from each other, but from ^‡.^

‡ not significantly different from each other, but from ^†.^

These figures reveal substantial variation in ^15^N enrichment between individual cells during the regular dark period. This variation can have diverse sources, including measurement error and temporal fluctuations in nitrogen fixation. However, given that the measurements are clearly above background (comparing Fig. 4A and Fig. 4B), and given the long duration of the incubation (3 hours), we conclude that this pattern likely reflects actual variation in activity between single cells, as for example reported in [19]. During the subjective dark, single-cell analysis provided insights that could not be gained from the bulk measurements. While the average ^13^C-DIC uptake and the average ^15^N_2_-fixation were both significantly larger than zero (Wilcoxon Signed Rank Test, p<0.0001), the two processes were negatively associated between cells: cells that showed high rates of photosynthesis showed low rates of nitrogen fixation, and vice versa (nonparametric test; Spearman’s Rho = −0.156; p (2-tailed) = 0.016). These results indicate that photosynthesis is mainly regulated by the availability of light, and nitrogen fixation by the circadian clock, and that both processes are thus triggered during the subjective dark period: The internal signal ‘scheduled’ the population for N_2_ fixation by increasing the necessary gene transcripts (Figure 2) and probably protein synthesis. On the other hand, the presence of light promoted photosynthesis and O_2_-production, generating a metabolic conflict with nitrogen fixation. While most cells in the population carried out photosynthesis under these conditions, some cells engaged mostly in N_2_ fixation, and attained rates of activity that were comparable to the rates achieved during the regular dark. That these cells had low rates of photosynthesis presumably allowed them to avoid inactivation of the nitrogenase by oxygen, and thus to circumvent the biochemical incompatibility of these two processes.

The molecular basis of the negative association between photosynthesis and nitrogen fixation at the single-cell level is currently not clear. One possibility is that the differential response of individual cells is due to differences in cellular capacities. Lechene and colleagues [22] suggested that such differences could explain individual N_2_ fixation rates. The study by Rust and colleagues [11] indicated that the circadian clock, and thus nitrogen fixation, is directly entrained by each cell’s energy metabolism. Stochastic differences in photosynthetic activities between cells could thus translate into slight differences in the phase of their circadian clock, and thus into different levels of nitrogen fixation. Another possibility could be the availability of iron for each individual cell. Saito *et al*. [30] showed that *C. watsonii* engages in “hotbunking” and recycles its iron from photosynthesis for N_2_ fixation and vice versa. This strategy reduces the cellular iron demand and could lead to a competitive advantage in iron-deplete oceanic regions [30]. However, the recycling would also enable the individual cells to only perform one of the two processes during the subjective dark period, as observed here. The fact that most cells engage in photosynthesis rather than N_2_ fixation could indicate that the former may be the metabolically more important process [31].

Stochasticity is only one of several causes for phenotypic heterogeneity (for review see [32], [33]). Ryall and colleagues [34] elaborated several aspects of phenotypic heterogeneity within populations with respect to a population response to a single environmental shift. This includes variation in growth rate, intracellular signals, age and size of cells, and external signals. Further, transcriptional responses can lead to bimodality, i.e. similar to an all-or-none response or the formation of subpopulations [34], [35]. Here, this could mean the formation of cells that carry out N_2_ fixation and others that engage in photosynthesis during the subjective dark period. Even though we did not detect changes in the relative transcript abundance of genes involved in N_2_ fixation and photosynthesis during the subjective dark period, any transcriptional shifts from a single population with medium transcript abundances to a population in a bimodal state may be covered up by the bulk measurement of transcripts via real-time quantitative PCR (RT-qPCR). Due to the pre-existing heterogeneity in N_2_ fixation and photosynthesis during the regular dark and light period, respectively, we think that the underlying stochasticity and the possible formation of bimodality during the subjective dark period (i.e. some cells engage in N_2_ fixation whereas the majority continues photosynthesis) are likely causes for the observed negative correlation between the two metabolically incompatible processes here.

Our approach allowed to directly measure metabolic activities of single cells, and thus to gain additional and more direct information about each cell’s state than by using transcriptional reporters. We observed large variation between cells in photosynthesis and nitrogen fixation (Figure 3 and 4). Additionally, and importantly, we find that these two processes were negatively correlated between cells in conditions where the circadian clock and the external signals do not match. Such negative associations between incompatible cellular activities can arise from simple regulatory circuits, for example from the proposed entrainment of light-driven metabolic states on the circadian clock. In the case of unicellular cyanobacteria, such correlated heterogeneity could potentially optimize a clone’s performance during transitions from light to dark: Single cells would switch rapidly from photosynthesis to nitrogen fixation, and thus avoid the costs of running incompatible processes in the same cell – but the moment of switching would be variable among individuals.

## Materials and Methods

### Experimental Setup

Axenic batch cultures of *Crocosphaera watsonii* WH8501 were grown in 2-L polycarbonate bottles at 28°C in phosphate and trace metal amended YBCII medium [36] without combined nitrogen in a 12∶12 h dark:light cycle with a light intensity of ∼ 70–100 µE m^−2^ s^−1^. The growth rate at the time of the experiment was 0.14 d^−1^ with cell densities ranging from 6.4 to 9.2×10^5^ cells ml^−1^. The experimental phase consisted of a 24 h phase under growth conditions followed by a 24 h continuous light phase. Cultures were kept in temperature- and light-controlled incubators with opposite dark:light regimes to facilitate sampling. Subsamples for stable isotope incubations and acetylene reduction assay to assess N_2_ fixation rates were taken every 4 and 3 h, respectively. Samples for gene expression analysis were taken every 4 h during the experimental phase. Cell abundance for calculations of cell-based rates was assessed at the beginning of each experimental dark or light phase using analytical flow cytometry. Subsamples for nanoSIMS analysis were taken from the stable isotope incubations at the end of the following incubation phases: the middle of the regular dark phase, the middle of the regular light phase, the middle of the subjective dark phase and the end of the continuous light phase.

### Acetylene Reduction Assay (ARA)

N_2_ fixation rates were assessed by incubating 3 ml of culture in 8.65 ml septum-capped vials containing 650 µl of acetylene in the headspace. Incubations lasted for ∼ 3 h and ethylene (C_2_H_4_) concentrations were then measured in a 250 µl headspace sample using a Shimadzu GC-14B gas chromatograph equipped with a flame ionization detector (FID) and a RT Alumina Plot column. C_2_H_4_ concentrations were calibrated with a dilution series ranging from 1 to 1000 ppm C_2_H_4_. C_2_H_4_ production was converted to N_2_ fixation with a conversion factor of 4∶1 (C_2_H_4_ produced:N_2_ fixed) [26].

### Stable Isotope Incubations

N_2_ and inorganic carbon fixation rates were determined by simultaneous incubation of *C. watsonii* with 280 µl ^15^N_2_ [37] and NaH^13^CO_3_ (1% of 2500 µmol L^−1^) in 100 ml glass serum bottles every 4 h during the 48 h experimental phase. Aliquots for elemental stoichiometry and bulk stable isotope analysis as well as for nanoSIMS (nanometer-scale secondary ion mass spectrometry) analysis were taken at the end of each ∼ 3-h incubation. Samples for bulk stable isotope analysis were filtered onto pre-combusted (450°C, 4 h) GF/F filters (Whatman), oven-dried (60°C for 6 h) and stored until analysis. Filters were pelletized in tin cups and analyzed using isotope ratio monitoring mass spectrometry. Samples for nanoSIMS were preserved with formaldehyde (1% (v/v) final) for up to 24 h at 4°C and subsequently filtered onto Au/Pd-sputtered GTTP filters (Isopore, 0.22 µm pore size, 25 mm). Filters were rinsed with sterile-filtered (0.2 µm) phosphate-buffered saline solution (PBS buffer), air-dried for 20 min and stored at −20°C until analysis.

### NanoSIMS Analysis

The NanoSIMS 50L (CAMECA) instrument was used for analysis. The sample surface was sputtered with a cesium primary ion beam with a current of 1–2 pA and an energy of 16 keV. The beam was focused to a nominal spot size of 130 nm. NanoSIMS working as an ion microprobe rastered the scanning area with the primary ion beam with a 256×256 pixel resolution and a dwelling time of 1 ms/pixel. Multiple scans were recorded for each area. Secondary ions extracted from each pixel of the sample surface were mass separated according to their mass to charge (m/z) ratio and counted in separated electron multiplier detectors. A mass resolving power of >7500 was used to separate secondary ions of the desired isotopes from mass interference from secondary ions with close m/z ratios. Two-dimensional images of the sample content were recorded for chosen ions (^12^C, ^13^C, ^12^C^14^N, ^12^C^15^N, ^31^P). Secondary electron images were recorded which provided information on surface topography. Regions of interest (ROI) were chosen around each individual cell. The isotopic ratios of individual cells were calculated for each ROI only taking ions originating from the cell into account.

### Calculation of N_2_ Fixation and Inorganic Carbon Uptake Rates

Biomass-specific N_2_ fixation and inorganic carbon uptake rates were calculated based on the atom percent of ^15^N and ^13^C in the particulate organic nitrogen or carbon (PON or POC) within either the bulk or the single-cell measurements. The ^15^N enrichment in the N_2_ pool was calculated from previous measurements of ^15^N_2_ concentration during a 3-hour incubation period under the same conditions [38]. Cellular rates were based on the bulk nitrogen and carbon content of the population divided by the total number of cells.

### Gene Expression Analysis

RNA extraction, cDNA synthesis and real-time quantitative PCR (RT-qPCR) were carried out as described previously [25]. Transcript levels of genes related to N_2_ fixation, photosynthesis and the cyanobacterial circadian clock were detected using *C. watsonii*-specific primers (Table 2). Transcript levels were calculated according to the 2^−ΔΔCt^ method [39], [40] and are presented as enrichment factor relative to the expression of *rpbI* (RNA polymerase) which was chosen as the calibrator.

**Table 2:** Primer sequences used in real-time quantitative PCR (RT-qPCR).

Target gene	CwatDraft #	Primer sequence (5′ –3′) F = forward, R = reverse
16S	R0029	F: CAT CAA ACC CAG CCT CAG TTC
		R: TTC ATG CTC TCG AGT TGC AGA
*kaiA*	4942	F: TGG CGA AGA TGC CGA CAT TA
		R: CGT CCA TCA GTT CCA TGT GCA
*kaiC*	4944	F: TCC ATC GAT TCG GTT ACT GCA
		R: GCG AAA AAT CTC CCG TCT CAC
*nifH*	3818	F: TGC TGA AAT GGG TTC TGT TGA A
		R: TCA GGA CCA CCA GAT TCT ACA CAC T
*ntcA*	0834	F: TGG TTC AAC CCG TGT GAC AGT
		R: TCT TGG CGT AAA TCT CCG AGA
P-II	5924	F: TCG CGG TTC GGA ATA TAC G
		R: AGC CCC TCT CAG CTT GTC AAT
*psaC*	5974	F: TTG CTT CCT CCC CTC GTA CA
		R: TTT CGC ATC GCT TAC AGC C
*psbA*1	1423	F: CTT CCT TCA ACA ACA GCC GTG
		R: CAG GCC ATG CAC CTA AGA AGA
*psbO*	4858	F: AAC ACC GGA ATT GCC AAC A
		R: TTG CAA GCA CAG ATC GTC AAC
*rbcL*	2714	F: CTT CCG CAT GAC TCC CCA
		R: TGC TGC ACC GGC TTC TTC
*rpb*1	3959	F: ACC GAA GCG GCT ATT GAA GGT
		R: TCC GGC AGG AAT CAA ACG A

The CwatDraft # of the permanent draft genome assembly (30 Jan 04, update 17 Oct 2007) is as reported on the Integrated Microbial Genomes website (http://img.jgi.doe.gov/cgi-bin/pub/main.cgi) provided by the Joint Genome Institute, U.S. Department of Energy.

### Statistical Analysis

Statistical analysis was performed using IBM SPSS Statistics 19.0.0 (SPSS Inc.).
